# The Interplay of Self-Construal and Service Co-Workers’ Attitudes in Shaping Emotional Labor Under Customer Injustice

**DOI:** 10.3390/bs15060735

**Published:** 2025-05-26

**Authors:** Yingkang Gu, Xiuli Tang

**Affiliations:** 1 School of Economics and Management, Shanghai Polytechnic University, Shanghai 201209, China; ykgu@sspu.edu.cn; 2 School of Event and Communication, Shanghai University of International Business and Economics, Shanghai 201620, China

**Keywords:** customer injustice, emotional labor, self-construal, service co-workers, experimental research method

## Abstract

Previous discussions on customer injustice and emotional labor have primarily focused on employee–customer dyads, often neglecting the role of service co-workers in shaping emotional labor dynamics. To address this gap, the current study integrates intrapersonal and interpersonal factors to explore their joint effects on employees’ emotional labor strategies when encountering customer injustice. A full-factorial experimental design with 2 (self-construal: independent vs. interdependent) × 3 (service co-workers: alone vs. positive attitudes vs. negative attitudes toward customer injustice) is employed, using data from 179 frontline service employees at high-star hotels in Shanghai, with self-construal and service co-workers operationalized as manipulated conditions. Results reveal that self-construal significantly influences surface acting: interdependent individuals are more inclined to engage in surface acting than independent individuals. By contrast, self-construal has no direct effect on deep acting. While service co-workers do not moderate the relationship between self-construal and surface acting, they play a critical role in the relationship between self-construal and deep acting: for interdependent employees, service co-workers’ attitudes (rather than their mere presence) decisively impact deep acting, with positive attitudes promoting deeper emotional engagement and negative attitudes reducing it. This study advances a dual-path framework highlighting how intrapersonal dispositions (self-construal) and interpersonal impression cues (service co-workers’ attitudes) interact to shape emotional labor. By expanding the traditional employee–customer dyad to a triadic model, the study bridges impression management theory and workplace injustice research, offering theoretical insights into how intrapersonal traits and interpersonal dynamics jointly shape contextualized emotional labor. This thereby provides a theoretical foundation for nuanced management strategies in service organizations.

## 1. Introduction

Customer injustice exists in a wide range of negative customer behaviors directed towards employees, including demeaning, disrespecting, and excessive demands, often occurring during the service interaction process. These behaviors increase employees’ negative emotions, leading to negative outcomes such as increased absenteeism, higher turnover rates (van Jaarsveld et al., 2015), and decreased service performance (Y. Park & Kim, 2019). Emotional labor, commonly defined as the rules of emotional display required by organizations during the service process, is a critical component of frontline employees’ daily work in service-oriented companies (Yang et al., 2019). Customer injustice behavior requires employees to exert higher levels of emotional labor and makes it more challenging for them to comply with the organization’s emotional display rules (Rupp & Spencer, 2006). Frontline employees, as agents of service interaction, play a crucial role in the production of service offerings, and their states and emotions directly affect emotional labor, which in turn influences service quality and customer satisfaction. However, compared to the emphasis on customers, the perceptual changes in emotions resulting from customer injustice and the corresponding coping strategies for such behaviors by frontline employees have not received sufficient attention.

In service interactions, service providers interact not only with customers but also with colleagues. Prior emotional labor studies have emphasized dyadic employee–customer interactions (Brotheridge, 2006; Grandey, 2000) and its impacts on employees (H. J. Kim, 2008; Rupp & Spencer, 2006), organizations, and co-consumption customers (J. Park & Kim, 2020), neglecting the triadic influence of service co-workers, defined as colleagues who jointly provide service and witness customer injustice. Service encounters usually involve a high level of social interaction (Markus & Kitayama, 1991), and individuals’ attitudes and behaviors are influenced by the other people in the scenario. When customer injustice occurs, it is possible that in addition to the customer and the employee, there may be other employees who jointly provide the service.

Compared to scenarios where an employee serves alone and encounters customer injustice, it remains unclear how the presence and attitudes of service co-workers towards customer injustice moderate the relationship between self-construal and emotional labor strategies in the context of customer injustice. Therefore, guided by self-construal theory (Markus & Kitayama, 1991), emotion regulation theory (Grandey, 2000) and impression management theory (Goffman, 1959), this study introduces “self-construal” as an intrapersonal variable that shapes employees’ cognitive interpretations of injustice, and “service co-workers” as an interpersonal variable that captures social normative influences to explore employees’ emotional labor across contexts. By integrating intrapersonal and interpersonal perspectives, this study addresses a gap in understanding emotional labor strategies under customer injustice, offering insights into how social contexts shape frontline employees’ adaptive responses.

## 2. Literature Review and Hypotheses

### 2.1. Customer Injustice

Skarlicki et al. (2008) pointed out that customer injustice predominantly reflected employees’ subjective experience, involving moral and social judgment factors, unlike customer aggression, which involved obvious anger displays. Xie et al. (2011b) proposed that customer injustice refer to disrespect or even harm exhibited by customers toward service employees through words, behaviors, or attitudes during the service process. Rupp and Spencer (2006) defined customer injustice primarily as interactional unfairness, including various unfair behaviors customers display toward employees during interactions. Based on this, van Jaarsveld et al. (2015) described customer injustice as unjust behaviors from customers, including interactional unfairness (lack of respect towards employees) and informational unfairness (lack of clear, open, or honest expression of service expectations).

Considering that customer information unfairness may be limited to certain roles where information sharing is expected between customers and employees, such as employees in the Complaint Handling Department (Holmvall & Sidhu, 2007), as well as the fact that among the various types of unfairness, interpersonal unfairness has the greatest impact on individuals’ emotions (Judge et al., 2006), this study therefore focuses on customer interaction unfairness, which captures employees’ perception of violating interpersonal justice by customers. These customer behaviors, such as making demanding requests that service employees cannot meet, deliberately belittling service employees’ work ability and performance, treating service employees in a condescending or patronizing manner, and being verbally abusive or mocking (Simillidou et al., 2020; Skarlicki et al., 2008), are all contrary to the social norm of mutual respect and are perceived as interpersonal injustice by employees.

Previous studies have found that employees experience a range of negative emotions following encounters with customer injustice behaviors, such as feeling aggrieved, angry, frustrated, worried, and fearful, which increases emotional labor stress (Xie et al., 2011a), reduces employees’ willingness to perform their job well, leads to emotional exhaustion and decreases service performance, and may even generate negative word-of-mouth from other customers within the same consumption context (Cai et al., 2018). Even a single episode of customer injustice behavior can trigger negative emotional experiences for employees, and considering the high frequency of such behaviors, the cumulative effects experienced by service personnel on a daily basis can have detrimental consequences. Compared to internal organizational unfairness, the unfairness from external customers is a more critical issue that requires greater attention.

### 2.2. Self-Construal and Employee Emotional Labor

Based on Markus and Kitayama’s self-construal theory, self-construal is a significant individual trait describing how individuals perceive and experience themselves in relation to others, encompassing two categories: independent and interdependent (Markus & Kitayama, 1991). Independent individuals tend to differentiate themselves from others and act based on their own thoughts with a stronger focus on autonomy, uniqueness, and individual goals, whereas interdependent individuals tend to connect themselves with others and social contexts, and their behavior is influenced by others with greater emphasis on relational harmony. Some studies have investigated how self-construal guides individuals’ thoughts, emotions, and behaviors through either connection or differentiation with others (Singelis, 1994; Wei et al., 2012).

Grandey introduced the emotion regulation theory, emphasizing that emotional labor was the process in which employees managed and regulated their emotional experiences and expressions to meet organizational requirements for emotions in the workplace (Grandey, 2000). Surface acting and deep acting are the most commonly used emotional labor strategies. Here, surface acting refers to employees regulating only the visible outward expressions of emotions without altering their genuine inner emotional experiences, while deep acting involves aligning inner emotional experiences with outward displays through emotional regulation (Tang & Gu, 2024). Both strategies enable employees to display positive emotions that align with organizational expectations. Numerous studies have explored the influence of individual characteristics on employee emotional labor, such as gender, age, personality traits (Dahling & Perez, 2010; Tang et al., 2017), etc. Self-construal is fundamentally about the relationship between self and others, and individuals with different self-construals exhibit varying degrees of attention to social situational factors (He et al., 2012). However, few studies have examined the relationship between self-construal and employee emotional labor.

Independent employees emphasize their “autonomy” independent of social context, focusing more on their own internal emotions and values, and tend to achieve self-worth in a more direct manner. In contrast, interdependent employees emphasize “harmony” in interpersonal relationships, valuing the thoughts, emotions, and behaviors of others. This difference in self-construal serves as the independent variable in our model, shaping employees’ cognitive interpretation of customer injustice: interdependent individuals may interpret customer injustice as a relational challenge (threatening interpersonal harmony), prioritizing surface acting to maintain harmonious interpersonal dynamics, while independent individuals view it as a personal stressor (challenging their autonomy), leading to deeper alignment of inner emotions with organizational expectations (deep acting). Customer injustice, as an antecedent of emotional labor demands, interacts with self-construal to shape employees’ strategy choices: independent individuals may interpret injustice as a personal stressor, while interdependent individuals perceive it as a shared challenge, influencing their reliance on surface or deep acting (Baranik et al., 2017). To comply with organizational rules regarding emotional expression, employees need to mobilize more psychological resources to fulfill their service responsibilities. Thus, in service contexts involving customer injustice, both independent and interdependent employees will experience varying degrees of psychological resource exhaustion. The conservation of resources theory suggests that individuals are motivated to maintain, protect, and acquire resources, while at the same time striving to preserve existing resources (Hobfoll, 1989). From the perspective of conservation of resources theory, deep acting enables independent employees to align their emotions with their autonomous values, thereby directly preserving self-efficacy, a core resource for their emotional stability, whereas surface acting may threaten their sense of internal consistency. In comparison, independent employees focus more on self-efficacy and are less influenced by others. When facing negative behavior from others, they exhibit lower levels of emotional fluctuation (Yeh & Hwang, 2011). Individuals with higher emotional stability are less likely to express inappropriate emotions and negative attitude towards unfairness compared to those with lower emotional stability (Milam et al., 2009). Therefore, independent employees experience less emotional labor change when encountering customer injustice.

However, for interdependent employees, customer injustice behavior creates high conflict with their ongoing quest for harmonious interpersonal relationships, making them more susceptible to negative emotions. Consequently, adhering to emotional expression rules becomes more challenging for them due to situational factors. Due to the impulsive nature of customer injustice behavior and the need for prompt service responses, interdependent employees may prefer surface acting as it requires less cognitive effort and psychological processes compared to deep acting, which may be more immediately feasible for them. Although there is no direct study on the relationship between employee self-construal and emotional labor, previous studies have indicated that employees with high susceptibility to negative emotion are more likely to engage in surface acting, while those with high susceptibility to positive emotion tend to engage in deep acting (Grant, 2013). Thus, we propose the following hypotheses:

**H1.**  *When encountering customer injustice, independent employees engage in less surface acting than interdependent employees.*

**H2.**  *When encountering customer injustice, independent employees engage in more deep acting than interdependent employees.*

### 2.3. Joint Effect of Self-Construal and Service Co-Workers

Social psychology research suggests that individual behavior in social interactions is influenced by the presence of others (Howard & Gengler, 2001). In service-oriented organizations, where production and consumption occur simultaneously, the service process is largely a face-to-face interaction. The behavior of service employees is significantly influenced by the presence of others (Wei et al., 2012). Anchored in Goffman’s (1959) impression management theory, which posits that individuals strategically align their behavior with social norms to meet others’ expectations, this study introduces “service co-workers” to explore the relational influence on employees’ emotional labor when encountering customer injustice.

Impression management, inherent in daily social interactions (Schlenker et al., 1996), involves individuals shaping favorable self-images. It is critical in service contexts where employees aim to influence perceptions of customers and colleagues (Zhao et al., 2019). This “expected image” motivation aligns with service employees’ pursuit of positive service images. From the perspective of impression management theory, colleagues’ positive and negative attitudes, as social norms within this framework, influence individuals’ self-presentation by motivating behavioral adjustments to foster desired impressions, especially for interdependent individuals attuned to relational harmony (Goffman, 1959). Conversely, independent individuals prioritize self-image consistency and act irrespective of situational cues, maintaining stable self-presentation across contexts. As independent employees are primarily guided by internal abilities, thoughts, and emotions rather than external norms, their emotional labor strategies remain consistent regardless of whether they serve alone or with others, and regardless of others’ attitudes toward customer injustice. Thus, we propose the following hypotheses:

**H3a.**  *When encountering customer injustice, service co-workers do not influence the surface acting of independent employees.*

**H3b.**  *When encountering customer injustice, service co-workers do not influence the deep acting of independent employees.*

In contrast, interdependent individuals, more concerned with situational factors like the presence of others (Lalwani & Shavitt, 2009), adjust their emotional labor in response to service co-workers’ attitudes toward customer injustice. Their behavior stems from a desire to conform to social norms and maintain relational harmony by avoiding adversarial conflict (Markus & Kitayama, 1991), a priority that shapes their self-presentation to align with group expectations rather than internal consistency. Grounded in considering others’ needs and seeking social approval during decision-making (Cross et al., 2000), this responsiveness to contextual cues reflects their distinct behavioral pattern, which emphasizes situational adaptation, unlike independent individuals who maintain stable self-presentation irrespective of external norms.

When encountering customer injustice, individuals with high job engagement are more likely to provide better customer service (Fine et al., 2010). Their enthusiasm for work and sense of responsibility can positively influence interdependent employees, offering additional psychological resources to regulate emotional labor. Although interdependent employees may initially resort to surface acting when facing customer injustice, the positive attitudes and behaviors when service co-workers can prompt them to reappraise the service situation, potentially shifting toward deep acting. While deep acting requires greater cognitive effort from employees, it reduces the depletion of psychological resources caused by emotional dissonance, the internal conflict between felt and displayed emotions (S. Xu et al., 2017). Conversely, when service co-workers hold a negative attitude toward customer injustice, the resulting unfair team atmosphere leads interdependent employees to feel disrespected, perceiving their role as merely satisfying customer needs (Holmvall & Sidhu, 2007). This intensifies psychological resource loss and increases the likelihood of surface acting. Thus, we propose the following hypotheses:

**H4a.**  *When encountering customer injustice, service co-workers influence the surface acting of interdependent employees.*

**H4b.**  *When encountering customer injustice, service co-workers influence the deep acting of interdependent employees.*

**Figure 1:**
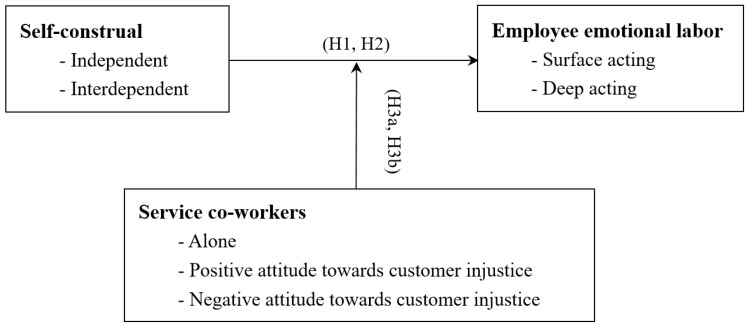
Conceptual framework.

In summary, the conceptual framework is shown in Figure 1.

## 3. Methodology

### 3.1. Experimental Design

This study employed a 2 (self-construal: independent vs. interdependent) × 3 (service co-workers: alone vs. positive attitudes vs. negative attitudes toward customer injustice) full-factorial experimental design. As noted by Chan and Wan (2008), this scenario-based approach is effective for exploring causal relationships, while simultaneously overcoming the memory-related biases and consistency challenges inherent in retrospective studies (Smith & Wagner, 1999) and enabling variable manipulation to support more robust causal inferences than retrospective methods (Bitner, 1990).

In this experimental design, self-construal was the independent variable, service co-workers acted as the moderating variable, and emotional labor was the dependent variable. Customer injustice was established as a uniform experimental premise. That is, all scenarios depicted identical customer injustice incidents, with variations limited to the presence and attitudinal orientations of co-service colleagues, which were merely designed to activate emotional labor needs without incorporating it as a variable in the model. Meanwhile, to operationalize the moderating variable, three between-subjects conditions were designed (alone/positive/negative), corresponding to the three levels of co-service colleague influence:

- Alone: When employees work independently, emotional labor strategies are driven by self-construal (interdependent vs. independent), free from social norm interference, as posited by self-construal theory (Markus & Kitayama, 1991). Independent individuals, prioritizing personal autonomy, rely more on surface acting to fulfill role requirements, whereas interdependent individuals, lacking relational cues, exhibit inconsistent emotional regulation due to the absence of social norm guidance.
- Positive: When employees work with service co-workers who hold a positive attitude toward customer injustice (e.g., maintaining a polite smile and using standard service phrases that align with company standards), these attitudes function as supportive social norms, consistent with self-construal theory’s emphasis on social norm adaptation (Markus & Kitayama, 1991). Interdependent individuals, attuned to relational expectations, reduce surface acting and engage in deep acting to maintain harmony, mitigating emotional inconsistency (Grandey, 2000).
- Negative: When employees work with service co-workers who hold a negative attitude (e.g., expressing acceptable dissatisfaction through eye-rolling or sighing), these attitudes acted as discouraging social cues, aligning with impression management theory’s focus on situational conformity (Goffman, 1959). Interdependent individuals, sensitive to relational tension, increased surface acting as a defensive strategy to navigate hostility, prioritizing external harmony over genuine engagement to adapt to the shared unfairness climate.

In summary, the experimental scenario settings of service co-workers, which range from no relational influence (alone) to supportive or discouraging norms (positive/negative), integrate self-construal and impression management theories, demonstrating how social norms shape self-presentation strategies rooted in individual self-construal.

Furthermore, to control for individual differences in perceived dissatisfaction with customer injustice (Ha & Jang, 2009), severity of customer injustice (Craighead et al., 2004), attributions to customer injustice (Chan & Wan, 2008), and authenticity of the experimental scenario (Maxham & Netemeyer, 2002; Wei et al., 2012), and to isolate the unique effects of self-construal and service co-workers on emotional labor strategies, these psychological constructs, as well as demographic factors such as gender and age, were included as control variables or covariates.

### 3.2. Samples

Shanghai, a pivotal economic and tourism hub in China with a highly competitive luxury hospitality sector, provides an ideal real-world context where service quality directly impacts brand reputation and customer loyalty. Frontline employees in these establishments face specific demands, such as delivering personalized service to meet exacting customer expectations, thus making them a representative sample to examine how colleagues and their attitudes (as social norms) shape emotional labor strategies when encountering customer injustice. Focusing on established downtown 4–5-star luxury hotels that upheld stringent service standards, had operated for at least five years, and featured over 300 rooms ensured the study’s empirical foundation was both contextually relevant and methodologically rigorous.

After obtaining approvals and support from the HR departments of the participating hotels, we recruited frontline employees, such as those at the front desk, concierge, and in the restaurant, based on a voluntary principle, and then carried out the experiment. Eventually, 197 frontline service employees participated in the experiment. Data from the 179 participants who completed the experiment and met data quality requirements were used in the analysis. Of the participants, 69 were male (*M_age_* = 22.217, *SD* = 0.820) and 110 were female (*M_age_* = 21.982, *SD* = 0.790).

### 3.3. Experimental Procedures

In Stage I, participants were seated and instructed to remain calm. After the researchers detailed the purpose and requirements, the participants were asked to complete a widely used 24-item self-construal scale developed by Singelis (1994).

In Stage II, participants were randomly assigned to one of three experimental scenarios. These scenarios were crafted based on a similar text used in a previous study (Wei et al., 2012) and incorporated the concept of customer injustice, along with items from existing measures. The scenarios were described as follows:

- Scenario 1—working alone towards customer injustice: You are a frontline employee at a high-star hotel in Shanghai, having worked there for years. One day, you are providing dining service for a table of customers alone. One of the customers is extremely picky about you, asks you in a sarcastic tone whether you are new and complains about your service, and even becomes angry with you at times. After the meal, they walk away arrogantly, without any response to your greeting of “thank you and welcome back again”, and do not even give you a direct look.
- Scenario 2—working with others who hold a positive attitude towards customer injustice: You are a frontline employee at a high-star hotel in Shanghai, having worked there for years. One day, you are providing dining service for a table of customers with your colleagues. One of the customers is extremely picky about you, asks you in a sarcastic tone whether you are new and complains about your service, and even becomes angry with you at times. After the meal, they walk away arrogantly, without any response to your greeting of “thank you and welcome back again”, and do not even give you a direct look. You know your colleagues hold a positive attitude towards such customer behaviors, maintaining a polite smile and using standard service phrases aligned with company standards.
- Scenario 3—working with others who hold a negative attitude towards customer injustice: You are a frontline employee at a high-star hotel in Shanghai, having worked there for years. One day, you are providing dining service for a table of customers with your colleagues. One of the customers is extremely picky about you, asks you in a sarcastic tone whether you are new and complains about your service, and even becomes angry with you at times. After the meal, they walk away arrogantly, without any response to your greeting of “thank you and welcome back again”, and do not even give you a direct look. You know your colleagues hold a negative attitude towards such customer behaviors, expressing subtle dissatisfaction through eye-rolling or sighing.

After reviewing the experimental materials, participants were asked to complete a questionnaire designed to conduct manipulation checks and measure study variables.

### 3.4. Measurements

A 7-point Likert scale was used for all questionnaires in this study.

Self-construal: It was measured using the self-construal scale developed by Singelis (1994), which includes 2 subscales (12 items each): the independent self-construal subscale assesses autonomy, individualism, self-centeredness, and behavioral consistency, while the interdependent self-construal subscale measures collective respect and relationship dependency. Subscale scores were averaged separately to derive independent and interdependent self-construal scores for each participant, with the higher score indicating their dominant self-construal (Singelis, 1994). Participants were categorized into groups based on this higher score: those with a higher independent self-construal score were assigned to the independent group (*N* = 72, *Cronbach’s α* = 0.820), and those with a higher interdependent score to the interdependent group (*N* = 107, *Cronbach’s α* = 0.792). Both subscales demonstrated acceptable reliability.

Emotional labor: Grandey’s Emotional Labor Strategy Scale was designed to capture the essence of intrinsic emotional experience and the alignment of external emotional expression during the emotional labor process (Grandey, 2003). This study drew on its conceptual framework and optimized the scale to enhance procedural efficiency and preserve the measurement validity of core constructs.

- Surface acting: A statement such as “When encountering such a customer, you would merely fulfill the service as required by the hotel, and even feign a smile” was used, measured via a 7-point semantic differential scale (anchored at “completely impossible”–“completely possible”, “not inclined to do this”–“inclined to do this”, and “definitely won’t do this”–“definitely will do this”) (Chan & Wan, 2008). In this study, it demonstrated acceptable reliability with *Cronbach’s α* = 0.845.
- Deep acting adopted a similar format: “When encountering such a customer, you can still strive to overcome your unpleasant emotions and serve the customer wholeheartedly with an amiable attitude”, which was also measured with the same 7-point semantic differential scale (Chan & Wan, 2008). In this study, it demonstrated acceptable reliability with *Cronbach’s α* = 0.891.

Service co-workers: A single-item measure was designed to validate the manipulation of “service co-workers” across scenarios. For scenario 1, the item stated, “You are serving the customer alone, without colleague involvement”. For scenario 2, the item stated, “The colleagues who provide service with you hold a positive attitude towards such customer unjust behaviors”. For scenario 3, the item stated, “The colleagues who provide service with you hold a negative attitude towards such customer unjust behaviors”.

Control variables and covariates: Gender and age were included as control variables to account for demographic influences. Meanwhile, dissatisfaction with customer injustice, severity of customer injustice, attributions to customer injustice, and authenticity of the experimental scenario were incorporated as covariates in the analysis model. Each construct addressed distinct dimensions of customer injustice perceptions across scenarios.

- Dissatisfaction: It represents employees’ emotional reaction to customer injustice. As a core affective response, it can confound the relationship between self-construal and strategy choice by amplifying or dampening emotional labor demands (Ha & Jang, 2009). Adapted from Chan and Wan (2008), it was measured with a three-item scale: “Overall, you are dissatisfied with this customer’s behaviors”, “Your overall experience with this customer’s behaviors is unpleasant” and “Overall, you are satisfied with this customer’s behaviors” (reverse-scored). The scale demonstrated good internal consistency with *Cronbach’s α* = 0.839.
- Severity: It was used to capture employees’ subjective assessment of the seriousness of customer injustice. As a core cognitive appraisal, it directly influenced employees’ emotional intensity and subsequent emotional labor strategy choices (Craighead et al., 2004). Adapted from Maxham and Netemeyer (2002), it was measured with a single item: “In your opinion, this customer’s behavior is very serious”.
- Attributions reflect employees’ judgments on whether the customer should be held responsible for the injustice. As a core accountability appraisal, they captured causal responsibility attributions critical for distinguishing internal (self-driven) vs. external (customer-driven) emotional labor motivations and influenced employees’ causal responsibility perceptions and emotional labor coping strategies (Wei et al., 2012). Adapted from Maxham and Netemeyer (2002), this construct was measured with three items: “The customer should take responsibility for the behaviors against you”, “You would blame the customer for such incidents”, and “The customer should take full responsibility for what you have suffered”. The scale demonstrated good internal consistency with *Cronbach’s α* = 0.844.
- Authenticity: It was used to capture employees’ subjective assessment of the realism of the entire experimental scenario. As a core cognitive appraisal, it directly influenced the priming of the experimental context (Wei et al., 2012). Adapted from Maxham and Netemeyer (2002), it was measured with a single item: “The situation described above is real”.

## 4. Results

Prior to analysis, the Shapiro–Wilk test was adopted to assess data normality. Results showed that all key continuous variables, including independent self-construal, interdependent self-construal, surface acting, and deep acting, exhibited a normal distribution (*p* > 0.05), indicating they met the normality assumptions required for subsequent analyses.

### 4.1. Manipulation Checks

To validate the effectiveness of the experimental scenario design, manipulation checks were first conducted for “self-construal”. The *t*-test results showed that participants in the independent group scored significantly higher on the independent self-construal subscale (*M_in_* = 5.450, *SD* = 0.674, *N* = 72; *t =* −8.268, *df* = 177, *p* = 0.000) compared to the interdependent self-construal subscale (*M_inter_* = 4.552, *SD* = 0.737, *N* = 107). In the interdependent group, participants scored significantly higher on the interdependent self-construal subscale (*M_inter_* = 5.079, *SD* = 0.663, *N* = 107; *t* = 4.393, *df* = 177, *p* = 0.000) compared to the independent self-construal subscale (*M_in_* = 4.613, *SD* = 0.740, *N* = 72). A significant between-group difference indicated that the self-construal grouping manipulation check was valid.

Second, manipulation checks were conducted for “service co-workers”. Results showed that participants in Scenario 1 acknowledged they were serving the customer alone (*M* = 5.672, *SD* = 0.926, *N* = 61); those in Scenario 2 acknowledged they were serving with colleagues who held a positive attitude toward customer injustice (*M* = 5.382, *SD* = 0.972, *N* = 55); and those in Scenario 3 acknowledged they were serving with colleagues who held a negative attitude toward customer injustice (*M* = 5.746, *SD* = 1.092, *N* = 63). Their means were substantially higher than the midpoint of four (on a seven-point scale), indicating that participants collectively perceived the preset presence and attitudinal orientations (positive/negative) of colleagues in the scenarios. This consistency confirmed that participants perceived their assigned scenarios as intended, validating the “service co-workers” manipulation.

Third, we intended to control for the potential effects of the severity and attribution of customer injustice on the results and to ensure the consistency of variables across the three experimental conditions. The ANOVA results confirmed that there were no significant differences among the three groups of participants in the perceived severity of customer injustice (*F_(2,176)_* = 0.554, *p* = 0.576, *η*^2^ = 0.006; *M_Alone_* = 4.623, *SD* = 1.635, *N* = 61; *M_Positive_* = 4.709, *SD* = 1.739, *N* = 55; *M_Negative_* = 4.921, *SD* = 1.495, *N* = 63), with pairwise comparisons showing small to negligible effect sizes (*Cohen*’*s d: alone* vs. *positive* = 0.051, *alone* vs. *negative* = 0.190, *positive* vs. *negative* = 0.131), and attributions to customer injustice (*F_(2,176)_* = 0.394, *p* = 0.675, *η*^2^ = 0.004; *M_Alone_* = 4.120, *SD* = 1.377, *N* = 61; *M_Positive_* = 3.903, *SD* = 1.163, *N* = 55; *M_Negative_* = 4.021, *SD* = 1.379, *N* = 63)*,* with pairwise Cohen’s *d* values ranging from 0.072 to 0.170 (all small effects by Cohen’s criteria) (Cohen, 1988). These results indicated that the participants’ perceptions of severity and attribution were almost identical across the three scenarios, confirming the consistency of variables under the three experimental conditions.

Last, regarding the authenticity of the experimental scenario, participants in the three conditions perceived it as realistic. The mean scores (*M_Alone_* = 5.934, *SD* = 1.153, *N* = 61; *M_Positive_* = 5.382, *SD* = 1.683, *N* = 55; *M_Negative_* = 5.683, *SD* = 1.268, *N* = 63) were substantially higher than the midpoint of four (*on a seven-point scale*). The ANOVA results showed no significant differences among the groups (*F*_(2,176)_ = 2.340, *p* = 0.099, *η*^2^ = 0.026), with pairwise Cohen’s *d* values ranging from 0.204 to 0.383 (small to medium effects by Cohen’s criteria) (Cohen, 1988), suggesting that scenario authenticity had no material effect on the results and remained stable across experimental conditions.

In sum, the above results indicated that participants perceived their respective situations precisely as intended, confirming the successful implementation of the experimental scenario manipulations and demonstrating a close alignment between the designed scenarios and participants’ perceptions.

### 4.2. Hypotheses Testing

#### 4.2.1. Effects of Self-Construal on Employee Emotional Labor

To examine the effects of self-construal and service co-workers on emotional labor strategies while controlling for demographic variables (gender and age) and psychological covariates (dissatisfaction, severity, attributions, and authenticity), this study employed a multivariate analysis of covariance (MANCOVA). Results revealed a significant main effect of self-construal on employees’ surface acting (*F*_(1,172)_ = 4.888, *p* = 0.028, *η*^2^ = 0.028).

Multiple comparisons (see Table 1) revealed that the interdependent participants (*M_inter_* = 4.968, *SD* = 1.231, *N* = 107) reported significantly lower levels of surface acting than the independent participants (*M_in_* = 5.355, *SD* = 1.048, *N* = 72; *t* = 2.260, *p* = 0.025), with a medium effect size (*Cohen*’*s d* = 0.332), supporting H1. The main effect of self-construal on employees’ deep acting was not significant (*F*_(1,172)_ = 0.266, *p* = 0.606, *η*^2^ = 0.002), and there was no significant difference in deep acting reported by independent and interdependent participants (*M_in_* = 3.349, *SD* = 1.509 vs. *M_inter_* = 3.546, *SD* = 1.811; *t* = −0.791, *p* = 0.430), with a negligible effect size (*Cohen*’*s d* = 0.120), not supporting H2.

#### 4.2.2. Moderating Effects of Service Co-Workers

Similarly, results by MANCOVA revealed that the main effect of service co-workers on employees’ deep acting was significant (*F*_(2,172)_ = 3.442, *p* = 0.034, *η*^2^ = 0.038), while the main effect of service co-workers on employees’ surface acting was not significant (*F*_(2,172)_ = 0.583, *p* = 0.559, *η*^2^ = 0.007), supporting H3a but not supporting H4a.

Further post hoc comparisons (see Table 2) revealed no significant difference in deep acting reported by independent participants when encountering customer injustice, compared to when they were alone or with service co-workers, regardless of whether the service co-workers’ attitude towards customer justice were positive (*M_Alone_* = 3.254, *SD* = 1.785, *N* = 21 vs. *M_Positive_* = 3.720, *SD* = 1.985, *N* = 25; *t* = −0.838, *p* = 0.407) with a small effect size (*Cohen*’*s d* = 0.246) or negative (*M_Alone_* = 3.254, *SD* = 1.785, *N* = 21 vs. *M_Negative_* = 3.615, *SD* = 1.696, *N* = 26; *t* = −0.705, *p* = 0.484) with a small effect size (*Cohen*’*s d* = 0.208). 

**Figure 2:**
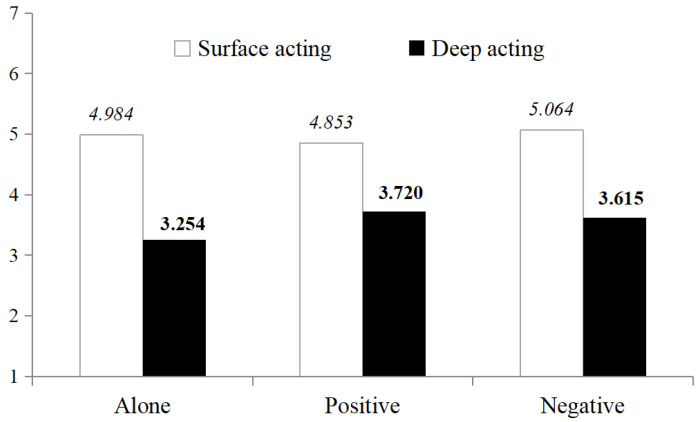
Employees’ deep acting influenced by service co-workers (independent group).

It indicates that neither the presence of service co-workers nor their attitude towards customer injustice (*M_Positive_* = 3.720, *SD* = 1.985, *N* = 25 vs. *M_Negative_* = 3.615, *SD* = 1.696, *N* = 26; *t* = 0.202, *p* = 0.840), with a negligible effect size (*Cohen*’*s d* = 0.057), would significantly influence the deep acting of independent employees. H3b was supported, which is shown in Figure 2.

Likewise, further post-hoc comparisons (see Table 3) revealed that when encountering customer injustice, interdependent individuals reported significantly lower levels of deep acting when alone (*M_Alone_* = 3.208, *SD* = 1.426, *N* = 40) compared to when serving with co-workers with a positive attitude towards customer injustice (*M_Positive_* = 4.089, *SD* = 1.838, *N* = 30; *t* = −2.178, *p* = 0.034), with a medium effect size (*Cohen*’*s d* = 0.546). However, there was no significant difference in deep acting reported by interdependent individuals when alone (*M_Alone_* = 3.208, *SD* = 1.426) compared to when serving with co-workers with a negative attitude towards customer injustice (*M_Negative_* = 2.901, *SD* = 1.048; *t* = 1.084, *p* = 0.282), with a small effect size (*Cohen*’*s d* = 0.246).

And in both cases where service co-workers were present, the interdependent individuals reported significantly higher levels of deep acting when serving with co-workers with a positive attitude towards customer injustice (*M_Positive_* = 4.089, *SD* = 1.838), compared to when serving with co-workers with a negative attitude towards customer injustice (*M_Negative_* = 2.901, *SD* = 1.048; *t* = 3.149, *p* = 0.003), with a big effect size (*Cohen*’*s d* = 0.817). It indicates that the attitude of service co-workers towards customer justice significantly influence the deep acting of interdependent employees, especially a positive attitude, which has a huge influence, partially supporting H4b.

**Figure 3:**
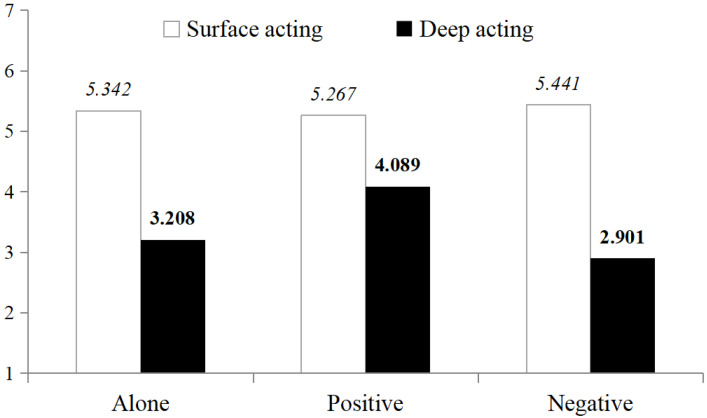
Employees’ deep acting influenced by service co-workers (interdependent group).

In all, the presence of service co-workers does not appear to be a key factor influencing the deep acting of interdependent employees; the main factor is the attitude of service co-workers towards customer injustice, particularly a positive attitude, which is shown in Figure 3.

## 5. Discussion

This study investigated the relationship between self-construal and employee emotional labor. When faced with customer injustice, self-construal exerted a significant influence on employees’ surface acting. Comparatively, interdependent employees were more inclined to engage in surface acting than independent employees. This indicates that interdependent individuals are more susceptible to the negative effects of customer injustice, which in turn affects their work attitudes and behaviors, supports the notion that self-construal can be regarded as an individual factor influencing employees’ emotional labor strategy choices and offers further support for the influence of self-construal on individuals’ cognitions, emotions, motivations, and behaviors (Wei et al., 2012) and enriches the research on individual characteristics among the factors affecting employee emotional labor.

However, in the context of facing customer injustice, self-construal did not significantly influence employees’ deep acting. This may be attributed to the triggering mechanism of deep acting, as deep acting necessitates that employees genuinely and internally endorse the service philosophy of “Customer First”, yet customer injustice directly challenges this endorsement. According to the conservation of resources theory (Ashforth & Humphrey, 1993), when individuals face a resource loss (such as psychological harm caused by customer injustice), they prioritize using general defensive mechanisms (e.g., emotional numbness) over relying on individual differences (like self-construal) to cope with stress. This process dilutes the influence of self-construal as situational pressure takes precedence. Additionally, Markus and Kitayama (1991) noted that individuals’ self-cognitive styles affected their emotion regulation strategies, but this effect became overshadowed in high-intensity customer injustice scenarios. The overwhelming situational stress may obscure the moderating role of self-construal, leading to its negligible impact on deep acting under such conditions.

In this study, no moderating effect of service co-workers in the context between self-construal and employees’ surface acting was found. Nevertheless, a moderating effect of service co-workers was detected in the context between self-construal and employees’ deep acting. When encountering customer injustice, self-construal exerted a significant influence on employees’ surface acting. However, when service co-workers were present, the relationship between self-construal and employees’ surface acting underwent a change. Everyone has an inherent need to present themselves favorably or to make a good impression on others, and this holds true in the service process, where individuals aspire to project the image as they desire to be perceived (Goffman, 1959). Deep acting is highly valued by organizations since the greater authenticity customers perceive in employees, the higher their satisfaction levels (Grandey, 2000). This demonstrates the professionalism and robust psychological qualities of service employees, namely, the “desired image” they strive to construct. Additionally, considering that employees spend more time with colleagues than with customers, they are more inclined to engage in deep acting when providing services jointly with colleagues. From the perspective of Resource Conservation Theory, colleagues also serve as a source of psychological resources for employees. When employees encounter customer injustice, positive attitudes are more likely to offer them emotional support and psychological resources. Therefore, service co-workers, as an important variable influencing employee emotional labor, deserve more attention from both scholars and practitioners.

For independent employees, when confronted with customer injustice, the attitude of service co-workers towards customer injustice, whether positive or negative, has no impact on their deep acting, because independent individuals typically have higher self-display goals aimed at expressing their individuality (Lalwani & Shavitt, 2009). In other words, their self-construal drives them to act in a manner consistent with their personal identity, regardless of the service co-workers’ attitude towards customer injustice. Conversely, for interdependent employees facing customer injustice, their level of deep acting hinges on the attitude of service co-workers. When service co-workers show a positive attitude towards customer injustice, interdependent employees are more likely to engage in deep acting compared to situations where service co-workers hold a negative attitude. This can be attributed to the fact that interdependent individuals place great importance on establishing and maintaining harmonious interpersonal relationships. They achieve this by emphasizing common ground with others. In the service context, when service co-workers are positive, it aligns with the interdependent employees’ goal of maintaining a positive team environment, thereby promoting deep acting. When service co-workers hold a negative attitude towards customer injustice, interdependent employees are likely to demonstrate a lower level of deep acting compared to when they provide service alone. This is because the behaviors of interdependent individuals are highly situation-dependent, especially in relation to service co-workers. A negative attitude from service co-workers may disrupt the harmonious environment they strive to maintain. As a result, when working alone, they can focus more on their own approach to service, which is often oriented towards the overall well-being of the customer interaction and are thus more likely to engage in deep acting.

## 6. Theoretical Contributions

This study explored employee emotional labor within the context of customer injustice by integrating self-construal theory (intrapersonal) and impression management theory (interpersonal). It proposed a dual-path framework that emphasized how individual self-concepts and social environmental cues collectively influenced emotional labor strategies. Through a 2 × 3 experimental design operationalizing self-construal and service co-workers, the study addressed a significant gap in existing research by examining the interplay between self-identity and social impression goals within employee–customer interactions. The theoretical contributions of this study are as follows:

First, in contrast to previous research mainly focused on the direct impact of customer injustice on employee emotional labor, this study employed a scenario-based experiment and introduced the “service co-workers” variable. By integrating self-construal (intrapersonal) and service co-workers (interpersonal) factors, it advanced emotional labor theory with a dual-path framework. This framework transcends the traditional “employee–customer” dyad, offering a more comprehensive view of how emotional labor emerges from the interplay between self-identity maintenance (self-construal) and social impression goals (service co-workers and their attitudes). Additionally, it validates and develops Grandey’s emotional regulation model by adding self-construal as an antecedent (Grandey, 2000), expanding the understanding of emotional labor.

Second, this study contributes to the refinement of impression management theory. While previous assumptions in impression management research implied that service co-workers’ mere presence could affect employees’ emotional regulation, especially deep acting, this study revealed that their attitude towards customer injustice was the critical moderator. This supplements the previous theory and emphasizes the importance of considering service co-workers’ attitudes when studying their influence on employees’ emotional labor, suggesting the boundary conditions of customer injustice’s impact.

Third, this study extended the research domain of employee emotional labor and workplace mistreatment. By revealing how service co-workers’ attitudes (as social impression cues) amplified or mitigated customer injustice effects, it bridged social psychology (self-construal as self-identity foundation) and organizational behavior (impression management as social adaptation), expanding the research scope from the traditional “employee–customer” dyad to a team-based triadic context where emotional labor serves both self-consistency (for independent individuals) and relational harmony (for interdependent individuals) goals. This finding implies that researchers should consider the interaction between individual self-concepts and social impression norms in addition to dyadic interactions, enriching the understanding of workplace injustice dynamics.

## 7. Managerial Implications

On the one hand, this study offers managers an alternative perspective on how interpersonal dynamics within work teams impact individuals when assisting employees in dealing with customer injustice. Employee emotional labor, which is trainable (Grandey, 2003) and malleable, is also influenced by social factors such as the number, hierarchy, and relationships among service co-workers in the work context. This study indicates that emotional labor is not merely a process through which employees convey positive emotions to customers but also a means of self-presentation driven by impression management needs. Due to such needs, even in the context of encountering customer injustice, the dynamic nature of employee emotional labor makes it possible to move from surface acting to the deep acting desired by organizations. Existing research has verified that social support can effectively reduce the psychological stress responses of individuals with an interdependent self-construal (Ren et al., 2019). Managers should acknowledge the importance of this social dynamic in aiding employees to manage customer injustice and in forming appropriate work teams to improve the efficiency of employee emotional labor.

On the other hand, when encountering customer injustice, employees, regardless of their independent or interdependent self-construal, may experience varying degrees of psychological resource depletion, leading to surface acting. Previous research has shown that employees experience internal tension during surface acting (S. T. Xu et al., 2020), which may give rise to negative consequences such as emotional exhaustion and job burnout (Krannitz et al., 2015). Service employees in service-oriented organizations are particularly susceptible to continuous emotional resource depletion. However, organizational care, as a critical work resource, can effectively mitigate employees’ negative behaviors and turnover intentions (Wen & Liang, 2020). Therefore, service-oriented organizations should attach equal importance to both customers’ needs and frontline employees’ emotions, and safeguard employees’ right to self-expression. Managers should prioritize two important aspects: one is to strengthen employee care mechanisms (e.g., providing timely organizational support and psychological counseling) (H. Kim & Qu, 2019) to address cumulative stress from customer injustice and prevent prolonged self-depletion; the other one is to design scenario-based training that incorporates service co-workers’ attitude cues (e.g., simulating colleague interactions with how to positively respond to customer injustice), so as to enhance employees’ psychological resilience and enhance their knowledge, flexible response skills, and emotional regulation capabilities in handling customer injustice.

## 8. Limitations and Future Research

This study has several limitations. First, the sample was exclusively drawn from hotels and primarily included young participants, which may limit the external validity of our findings. Future research should test generalizability across diverse service industries (e.g., retail, healthcare) and workplace contexts. Second, although we accounted for key covariates, the observational design made it challenging to account for all potential variables (e.g., job tenure, customer service training, personality traits, cultural background), which could introduce unmeasured confounds and affect internal validity. These factors warrant closer examination in future studies. Third, contrary to theoretical expectations, self-construal did not influence deep acting, and colleagues’ characteristics did not moderate the link between self-construal and surface acting. This suggests that less explored contingency factors, such as emotional regulation capacity or organizational norms, may influence these relationships, necessitating theory-driven investigations into boundary conditions. Considering these gaps, future research could incorporate social network theories (Cao & Xiao, 2014) to explore how the structure of colleague relationships (e.g., hierarchy, relational ties, team size) affects emotional labor strategies during customer injustice. Such research would enhance the comprehension of how social contexts interact with self-construal to motivate adaptive behaviors in service settings.

## Figures and Tables

**Table 1:** Means and standard deviations of dependent variables by self-construal.

Dependent Variable	Self-Construal	*N*	*M*	*SD*	*t*	*p_(2-tailed)_*	*Cohen’s d*
Surface acting	Independent	72	5.355	1.048	2.260	0.025	0.332
	Interdependent	107	4.968	1.231			
Deep acting	Independent	72	3.546	1.811	−0.791	0.430	0.120
	Interdependent	107	3.349	1.509			

**Table 2:** Means and standard deviations of employees’ deep acting (independent group).

Service Co-Workers	*N*	*M*	*SD*	*t*	*p_(2-tailed)_*	*Cohen’s d*
Along	21	3.254	1.785	−0.838	0.407	0.246
Positive	25	3.720	1.985			
Along	21	3.254	1.785	−0.705	0.484	0.208
Negative	26	3.615	1.696			
Positive	25	3.720	1.985	0.202	0.840	0.057
Negative	26	3.615	1.696			

**Table 3:** Means and standard deviations of employees’ deep acting (interdependent group).

Service Co-Workers	*N*	*M*	*SD*	*t*	*p_(2-tailed)_*	*Cohen’s d*
Along	40	3.208	1.426	−2.178	0.034	0.546
Positive	30	4.089	1.838			
Along	40	3.208	1.426	1.084	0.282	0.244
Negative	37	2.901	1.048			
Positive	30	4.089	1.838	3.149	0.003	0.817
Negative	37	2.901	1.048			

## Data Availability

The datasets analyzed in this study are available from the corresponding author upon reasonable request.
